# Effect of Addition of Taurine on the Liquid Storage (5**°**C) of Mithun (*Bos frontalis*) Semen

**DOI:** 10.1155/2013/165348

**Published:** 2013-06-15

**Authors:** P. Perumal, Kezhavituo Vupru, C. Rajkhowa

**Affiliations:** Animal Reproduction Laboratory, National Research Centre on Mithun (ICAR), Jharnapani, Nagaland 797 106, India

## Abstract

The present study was undertaken to assess the effect of taurine on sperm motility, viability, total sperm abnormalities, acrosomal and plasma membrane integrity, enzymatic profiles such as reduced glutathione (GSH), glutathione peroxidase (GPX), superoxide dismutase (SOD), and catalase (CAT), and biochemical profiles such as cholesterol efflux and malondialdehyde (MDA) production. A total of 50 ejaculates were collected twice a week from 8 mithun bulls, and semen was split into 4 equal aliquots and diluted with the TEYC extender. Group 1: semen was without additives (control); groups 2 to 4: semen was diluted with 25 mM, 50 mM, and 100 mM of taurine, respectively. Seminal parameters and enzymatic and biochemical profiles were assessed at 5°C. Inclusion of taurine into diluent resulted in significant (*P* < 0.05) decreases in percentages of dead spermatozoa, abnormal spermatozoa, and acrosomal abnormalities after liquid storage compared with the control group. Additionally, taurine at 50 mM has significant improvement in quality of mithun semen than taurine at 25 or 100 mM stored in *in vitro* at 5°C. It was concluded that the possible protective effects of taurine on sperm parameters are from enhancing the function of antioxidant enzymes, preventing efflux of cholesterol from cell membranes and decreased MDA production.

## 1. Introduction

Mithun (*Bos frontalis*) is a semiwild free-ranging, rare bovine species present in the North-Eastern Hill (NEH) region of India. It is believed to have originated more than 8000 years ago from the wild Indian gaur (*Bos gaurus*) [1]. The animal has an important place in the social, cultural, religious, and economic life of the tribal population, particularly in the states of Arunachal Pradesh, Nagaland, Manipur, and Mizoram [2]. Recent statistics indicate that the mithun population is decreasing gradually due to lack of suitable breeding bulls, increased intensive inbreeding practices, declining land area for grazing, lack of suitable breeding, and feeding management. Greater efforts are required from all quarters to preserve the mithun population to enhance the socioeconomic status of this region. Since mithuns are not fully domesticated, natural breeding is practiced in this species with accompanied limitations like cost and disease transmission. Thus, use of artificial insemination for improvement is essential.

 Cold storage of semen is used to reduce metabolism and maintain sperm viability over an extended period of time. Research into extender development has focused on membrane stabilizing compounds, antioxidants, and cryoprotectants. It was reported that fresh mithun semen can be preserved successfully at 5°C for 3 days using a tris-egg yolk-based extender without glycerol [3]. But the quality of semen deteriorated during this extended storage. One cause of this decline is due to the action of the reactive oxygen species (ROS) generated by the cellular components of semen, namely, superoxide anion radical (O_2_^−^) and hydrogen peroxide (H_2_O_2_) [4, 5]. Moreover, bovine spermatozoa are prone to membrane damage due to high content of polyunsaturated fatty acids in the sperm membrane and lack of a significant antioxidant system in the cytoplasm [6]. This makes bovine spermatozoa particularly susceptible to lipid peroxidation (LPO) in presence of ROS [7]. Bansal and Bilaspuri [8] recently reviewed the impact of oxidative stress and antioxidants on sperm function and explained effects of various types of antioxidants. The effects of lipid peroxidation include irreversible loss in motility, damage to the sperm DNA, and decreased fertility [4, 9]. In recent years, studies have also been conducted on bovine semen diluents, including additives such as, taurine, trehalose, selenium, GSH, GPX, CAT, SOD, and surfactant compounds, so as to improve motility, viability, and membrane integrity of spermatozoa [10, 11].

 The addition of antioxidants such as taurine to ovine sperm [12], feline sperm [13], and rabbit sperm [14] has been shown to protect sperm against the harmful effects of ROS and improve sperm motility and membrane integrity during sperm liquid storage. Taurine is sulfonic amino acid and is nonenzymatic scavenger that plays an important role in protection of spermatozoa against ROS, in case of exposure to aerobic conditions and storage at 4°C under refrigerator [12–14]. In recent years, taurine has been used as anti-oxidant in semen extenders and has been used in the cryopreservation of boar [15], bull [16], human [17], ram [18], and goat sperm [19] to improve motility, viability, membrane integrity, and fertility of spermatozoa by inhibiting lipid peroxidation and protecting cells against accumulation of ROS [20, 21]. Further, perusal of the literatures revealed no information on the effect of addition of taurine on maintenance of sperm viability during low temperature liquid storage of mithun semen. Hence, the objective of this study was to assess the effect of this additive on the seminal parameters, MDA production, cholesterol efflux, and antioxidant profiles such as GSH, GPX, SOD, CAT, and total antioxidant capacity (TAC) of mithun semen.

## 2. Material and Methods

### 2.1. Animals and Semen Collection

Eight apparently healthy mithun bulls, approximately 4 to 6 years of age, were selected from the herd derived from various hilly tracts of the NEH region of India. The average body weight of the bulls was 501 kg (493 to 507 kg) at 4 years, which increased to 530 kg (523 to 538 kg) at 6 years of age with good body condition (score 5-6) maintained under uniform feeding, housing, and lighting conditions at the National Research Centre on Mithun, Jharnapani, Nagaland, India, which lies at 25°54′30′′ North Latitude and 93°44′15′′ East Longitude at an altitude range of 250–300 MSL. Each experimental animal was offered ad libitum drinking water and 30 kg mixed jungle forages (18.4% dry matter and 10.2% crude protein) and 4 kg concentrates (87.1% dry matter and 14.5% crude protein) fortified with mineral mixture and salt daily. Semen was collected from the animals by rectal massage. Oxytocin (5 IU, intramuscular) was injected just prior to rectal palpation. Briefly, seminal vesicles were massaged centrally and backwardly for 5 min followed by the gentle milking of ampullae one by one for 3–5 min, which resulted into erection and ejaculation. During collection, the initial transparent secretions were discarded, and neat semen drops were collected in a graduated test tube with the help of a funnel. During the study, all the experimental protocols met the Institutional Animal Care and Use Committee regulations.

### 2.2. Semen Processing and Evaluation

A total of 50 ejaculates were collected via rectal massage from the mithun twice a week from the experimental animals, and semen was pooled to eliminate individual differences. Immediately after collection, the samples were kept in a water bath at 37°C and evaluated for volume, colour, consistency, mass activity, and pH. After the preliminary evaluations, samples were subjected to the initial dilution with prewarmed (37°C) Tris egg yolk citrate extender (TEYC) (tris-hydroxymethyl aminomethane 3.028% (w/v), sodium citrate 1.655% (w/v), fructose 1.250% (w/v), and egg yolk 20% (v/v); 100,000 IU penicillin G (sodium salt) and 100 mg dihydrostreptomycin were added in 100 mL of buffer). The partially diluted samples were then brought to the laboratory in an insulated flask containing warm water (37°C) for further processing. The ejaculates were evaluated and accepted for evaluation if the following criteria were met: concentration: >500 million/mL, mass activity: >2.5+, individual motility: >70%, and total morphological abnormalities: <10%. 

Each pooled ejaculate was split into four equal aliquots and diluted with the TEYC extender with taurine. Group 1: semen was without additives (control); groups 2 to 4: semen, with 25 mM, 50 mM, and 100 mM of taurine, respectively at a final concentration of 25 × 10^6^ spermatozoa per mL. However, pH of diluents was adjusted to 6.8–7.0 by using phosphate buffered solution. Sperm concentrations were determined with the aid of a haemocytometer [22]. Diluted semen samples were kept in glass tubes and cooled from 37 to 5°C, at a rate of 0.2-0.3°C/min in a cold cabinet and maintained at 5°C during liquid storage for the experiment. The percentage of sperm motility, viability, total sperm abnormality, acrosomal integrity, and plasma membrane integrity by hypo-osmotic swelling test (HOST) was determined as per standard procedures in samples during storage of semen at 5°C. Sperm motility was assessed by analyzing four to five fields of view of sample placed on a prewarmed slide (37°C) under prewarmed cover slip (37°C) using bright-field optics (Nikon, Eclipse 80i; magnification 400x). Before determination of progressive motility, the stored samples were warmed in a water bath at 37°C for 5 min.

The count of live spermatozoa was determined using eosin-nigrosin stain 5% (w/v) nigrosin water soluble, 0.6% (w/v) eosin yellow water soluble, and 3% sodium citrate dehydrate, filtered and pH adjusted to 7.0 by adding few drops of 0.1 M NaH_2_PO_4_ or 0.1 M Na_2_HPO_4_ according to a previously described method [23] using bright-field optics (Nikon, Eclipse 80i; magnification 1000x). Spermatozoa (eosin-nigrosin stained; 200 per sample) were also evaluated under bright-field optics (Nikon, Eclipse 80i; magnification 1000x) for morphological abnormalities. Acrosomal integrity was assessed by Giemsa staining as described by Watson [24]. 

The HOST was used as a complementary test to the viability assessment protocol to evaluate the functional integrity of the sperm plasma membrane. HOST relies on the resistance of the membrane to loss of permeability under stress condition of swelling in a hypo-osmotic medium [25]. Sperm cells with resistant membranes exhibited swelling around the tail such that the flagella become curled, and the membrane maintained a swollen bubble around the curled flagellum. The assay was performed by mixing 30 *μ*L of semen with a 300 *μ*L 100 mOsm/kg hypo-osmotic solution (9 g fructose plus 4.9 g sodium citrate per liter of distilled water) [26]. This mixture was incubated (37°C) for 1 h, and 0.2 mL of the mixture was placed on a microscope slide and mounted with a cover slip and immediately evaluated (Nikon, Eclipse 80i; 400× magnification) under a phase-contrast microscope. A total of 200 spermatozoa were counted in at least five different microscopic fields. The percentages of sperm with swollen and curled tails were then recorded.

Antioxidant profiles such as GSH, GPX, CAT, SOD, TAC, and biochemical profiles such as total cholesterol, glucose-6-phosphate dehydrogenase (G6PD or G6PDH), aspartate amino transaminase (AST), and alanine amino transaminase (ALT) were estimated by commercially available diagnostic kits, whereas LPO of sperm and seminal plasma was measured by determining MDA production, using thiobarbituric acid (TBA) as per the method of Buege and Aust [27] and modified by Suleiman et al. [28]. 

### 2.3. Statistical Analyses

Results were analyzed statistically and expressed as the mean ± SEM. Means were analyzed by one way analysis of variance, followed by the Tukey's post hoc test to determine significant differences between the four experimental groups, that is, with additives or no additive on the sperm parameters using the SPSS/PC computer program (version 15.0; SPSS, Chicago, IL). Differences with values of *P* < 0.05 were considered to be statistically significant after arcsine transformation of percentage data by using SPSS 15. 

## 3. Results

**Figure 1:**
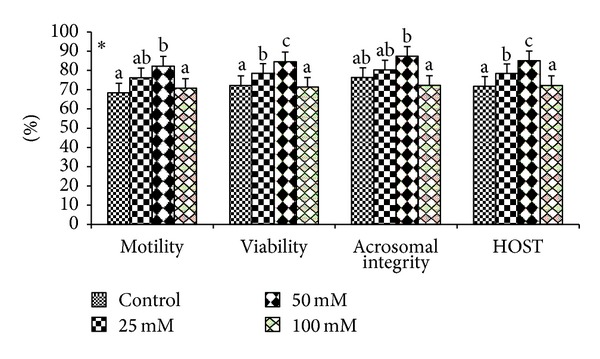
Effect of diluent supplementation with taurine on seminal parameters of mithun semen (* indicates *P* < 0.05).

**Figure 2:**
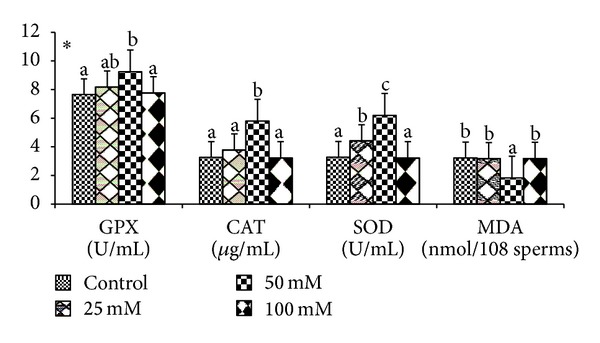
Effect of diluent supplementation with taurine on GPX, CAT, SOD, and MDA production in mithun semen (* indicates *P* < 0.05).

**Figure 3:**
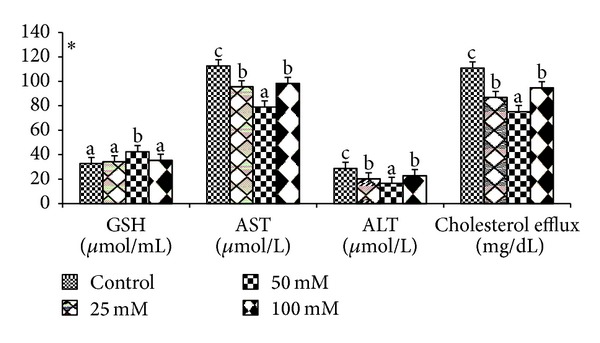
Effect of diluent supplementation with taurine on GSH, AST, ALT, and cholesterol efflux in mithun semen (* indicates *P* < 0.05).

**Figure 4:**
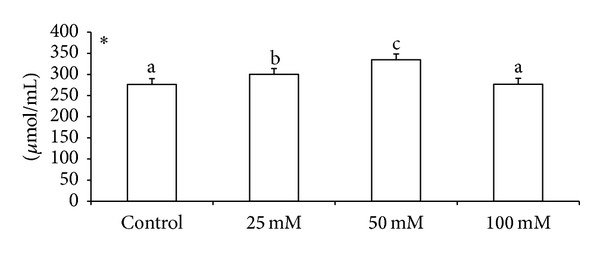
Effect of diluent supplementation with taurine on TAC in mithun semen (* indicates *P* < 0.05).

**Figure 5:**
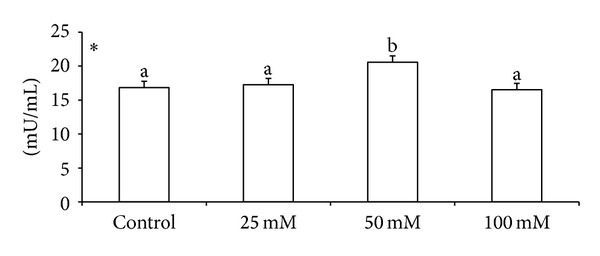
Effect of diluent supplementation with taurine on G6PDH in mithun semen (* indicates *P* < 0.05).

Effects of various doses of taurine on sperm motility, viability, and acrosomal and plasma membrane integrity in liquid storage (5°C) are presented in Figure 1. Results revealed that inclusion of taurine into diluent resulted in decreases (*P* < 0.05) in percentages of dead spermatozoa, abnormal spermatozoa, and acrosomal abnormalities when semen samples were examined at different hours of storage periods compared with the control group. Averaged over time, mean total sperm abnormalities were 8.38 ± 0.26, 7.84 ± 0.74, 5.75 ± 0.32, and 7.62 ± 1.23, respectively, for control, 25 mM, 50 mM, and 100 mM of taurine-treated mithun semen. Additionally, taurine at 25 and 100 mM were inferior to taurine 50 mM treatments for these characteristics, and there was a significant (*P* < 0.05) difference between taurine at 25 and 100 mM for these response. The antioxidant enzymatic profiles revealed that highest mean SOD and CAT, GSH (Figure 2), GPX activity, TAC (Figure 4), and G6PDH (Figure 5) were recorded in taurine treated semen than control group and differed (*P* < 0.05) between groups. Intracellular enzymes such as AST, ALT (Figure 3) decreased (*P* < 0.05) in taurine treated semen. Similarly cholesterol efflux (Figure 3) and MDA production (Figure 2) differed significantly between the taurine treated and control and were lower in the taurine treated group. It was obvious from the data of this experiment that addition of taurine, especially at 50 mM, to the semen diluent resulted in significant improvement in quality, antioxidant enzymatic activity, and reduction of cholesterol efflux and MDA production of mithun semen stored *in vitro* at 5°C.

## 4. Discussion

In the present study, the results revealed that addition of taurine has improved the seminal parameters, antioxidant and biochemical profiles of mithun semen, and thus it protects the structures and functions of spermatozoa efficiently. Thus, it may enhance the quality of semen by preserving efficiently during artificial insemination procedure. To the best of our knowledge, this is the first report of the effect of taurine on seminal parameters, antioxidative enzymatic and biochemical profiles in mithun semen. Analysis of various seminal parameters such as forward progressive motility, livability, and acrosomal and plasma membrane integrity are important for extensive utilization of semen in artificial insemination, and these parameters revealed significant difference between the treatment groups. The beneficial effects of taurine in the semen preservation are due to that it is a very potent antioxidant [29]. The sulfonated amino acid, taurine, present in both epididymal and oviduct fluid, is an important protector of cells against the accumulation of ROS when exposed to aerobic conditions [14, 30]. In the present study, effect of taurine on these parameters were similar to results obtained by other researchers, who observed supplementation of taurine to protect unfrozen rabbit [14], ram [12], and frozen-thawed ram sperm [11].

 Because the mammalian sperm membrane has high polyunsaturated fatty acids, it renders the sperm very susceptible to LPO, which occurs as a result of the oxidation of the membrane lipids by partially reduced oxygen molecules, such as O_2_^−^ and H_2_O_2_. Lipid peroxides impair the sperm function through altered sperm motility, membrane integrity, and damage to sperm DNA and fertility through oxidative stress and production of cytotoxic aldehydes [31]. In addition, the antioxidant system of seminal plasma and spermatozoa is compromised during semen processing [32]. Therefore, inclusion of exogenous antioxidants may modulate the antioxidant system of semen.

The results of the present study were showed that addition of 50 mM taurine improves keeping quality of mithun semen preserved at 5°C. The sperm motility was declined by the time of storage and remained over 50% for up to 30 hours. In contrast, decline rate in the motility percentage was higher in semen samples treated with 100 mM taurine or without taurine. It has been reported that the quality of chilled semen decreased with time and remained suitable for use up to 30 hours as judged by motility and morphology [3, 33]. The improvement of semen quality by addition of exogenous taurine recorded in the present study was previously reported in bull semen in the form of motility and intact acrosomal membrane [34]. Moreover, 50 mM taurine was significantly improving the percentages of sperm viability and intact plasma membrane [29].

 Taurine helps maintaining the integrity of normal acrosome [34] and stabilizes the plasmalemma of spermatozoa and so increases motility. Taurine, in sperm cells, is able to react with many reactive oxygen species directly for protecting mammalian cells against oxidative stress, and hence maintaining sperm motility [18]. Therefore, as seen by this study, attempts to improve the motility and viability of the sperm cells by incorporating taurine in liquid storage [13, 14, 27] and frozen semen form [29].

 Moreover, it maintains plasma, mitochondrial membrane integrity, and cytoskeleton structure of flagella of sperm as cell protecting effects. Taurine also protects SOD and CAT level in the semen extender [18], which helps to maintain membrane transportation and fertility of the spermatozoa. The axosome and associated dense fibers of the middle pieces in sperm are covered by mitochondria that generate energy from intracellular stores of ATP. These are responsible for sperm motility [35]. Based on our results, we can hypothesize that additive taurine displayed protective effects on the functional integrity of the axosome and mitochondria, improving sperm motility in liquid storage of mithun semen.

 AST and ALT are essential for metabolic processes which provide energy for survival, motility, and fertility of spermatozoa, and these transaminase activities in semen are good indicators of semen quality because they measure sperm membrane stability [36]. Thus, increasing the abnormal spermatozoa in liquid storage causes high concentration of transaminase enzyme in the extracellular fluid due to sperm membrane damage and ease of leakage of enzymes from spermatozoa [37]. Moreover, decrease in AST and ALT activities of seminal plasma and semen in taurine treated semen may be due to that it maintains structural stability of the sperm [38]. In the present study, AST and ALT levels were lower in taurine treated semen as it stabilizes the membrane integrity of acrosome, plasma, mitochondria, and flagella of the sperm. 

 Glutathione (L-g-glutamyl-L-cysteinylglycine; GSH) is the most abundant nonprotein thiol in mammalian cells and is present mainly in reduced form (GSH), and only a small amount is in oxidized form (GSSG). GSH antioxidant system consists of reduced GSH, oxidized GSSG, glutathione reductase (GRD), GPX, and glutathione-s-transferase. GRD stimulates the reduction of GSSG to GSH. This ensures a steady supply of the reductive substrate (NADPH) to GPX. G6PD is required for the conversion of NADP to NADPH, which is called as GSH oxidizing-reducing cycle in sperm and seminal plasma. In the present study, GSH and GPX were higher in the seminal plasma of taurine added semen [9, 18, 34] as they maintain the antioxidant system in liquid storage of mithun semen.

 Catalase is a tetramer of 4 polypeptide chain antioxidant which is found in nearly all living organisms exposed to oxygen. It is derived from the epididymis, seminal vesicle, and detoxifies both intra- and extracellular H_2_O_2_ by reduction to H_2_O and O_2_ [39], and it also prevents the loss of motility caused by ROS generated by leukocyte in the semen [40]. Similarly, SOD catalyzes dismutation of superoxide into oxygen and hydrogen peroxide. It scavenges both extracellular and intracellular superoxide anion and prevents lipid peroxidation of the plasma membrane. SOD spontaneously dismutase (O_2_^−^) anion to form O_2_ and H_2_O_2_. SOD also prevents premature hyperactivation and capacitation induced by superoxide radicals before ejaculating [41]. In the present study, the concentration of SOD and CAT was higher in taurine treated semen. But normally, seminal plasma is a potent source of this antioxidant, SOD [42]. The high levels of readily peroxidizable polyunsaturated material expose spermatozoa to excessive oxidative stress, and the SOD activity of sperm samples is a good predictor of their survival time. Taurine, when applied at a dose of 50 mM, improved sperm motility during preservation and displayed antioxidative properties, elevating the CAT level, in association with SOD concentration, similar to a study carried out in rabbit [14] and bull semen [34]. Further, taurine, a permeating cryoprotectant, acts as an antioxidant and causes membrane lipid and protein rearrangement, which results in increased membrane fluidity, greater dehydration at lower temperatures and therefore increased ability of spermatozoa to survive during this preservation [43]. This could be one of the reasons for improved motility, viability, and membrane integrity of spermatozoa, diluted in presence of taurine in the semen extender.

G6PD or G6PDH is a cytosolic enzyme in the pentose phosphate pathway, a metabolic pathway that supplies reducing energy to cells by maintaining the level of the coenzyme nicotinamide adenine dinucleotide phosphate (NADPH). NADPH in turn maintains the level of glutathione in these cells that helps to protect the cells against oxidative damage. In the present study, the G6PDH level was higher in the taurine treated semen as indicates it increases the function of antioxidant glutathione and glucose/fructose utilization by the sperm cells leads to good semen quality [44] and is crucial for sperm-fertilizing ability [45].

Taurine prevents efflux of cholesterol from the sperm membrane and MDA production in diluents which indicates it prevents premature capacitation and acrosomal reaction that act as an antioxidant. Along with phospholipids, cholesterol is necessary for cell physical integrity and ensures fluidity of the cell membrane. Cholesterol plays a special role in the sperm membrane because its release from the sperm membrane initiates the key step in the process of capacitation and acrosome reaction that is crucial for fertilization [46]. Moreover, adding cholesterol to diluents prior to defreezing increases sperm resistance to stress caused by the freezing-defreezing procedures, preserving sperm motility and fertilization potential [47]. In the present study, the efflux of cholesterol and MDA production were decreased in treated group as compared to the control untreated group [18, 29, 34, 48]. So, the semen samples treated with taurine will have high cryoresistance power than untreated control group. In the present study, it was observed that sperm parameters that received at 50 mM of taurine were significantly higher than those of the other and control group. 

In this study, improvements observed in sperm quality may be attributed to prevent excessive generation of free radicals, produced by spermatozoa themselves, by means of their antioxidant property of taurine. It was concluded that the possible protective effects of taurine supplementation enhance the antioxidant enzyme content and prevent efflux of cholesterol and phospholipids from cell membrane and MDA production. Thus, it may protect the spermatozoa during preservation and enhance the fertility in this species. Future sperm preservation/cryoprotective studies are warranted to confirm the present findings.
